# Essential Role of Zinc and Zinc Transporters in Myeloid Cell Function and Host Defense against Infection

**DOI:** 10.1155/2018/4315140

**Published:** 2018-10-17

**Authors:** Muna Sapkota, Daren L. Knoell

**Affiliations:** Department of Pharmacy Practice, College of Pharmacy, 86120 Nebraska Medical Center Omaha, NE 68198-6120, USA

## Abstract

Zinc is an essential micronutrient known to play a vital role in host defense against pathogens. Diets that are deficient in zinc lead to impaired immunity and delayed recovery from and worse outcomes following infection. Sustained insufficient zinc intake leads to dysregulation of the innate immune response and increases susceptibility to infection whereas zinc supplementation in at-risk populations has been shown to restore host defense and reduce pathogen-related morbidity and mortality. Upon infection, zinc deficiency leads to increased pathology due to imbalance in key signaling networks that result in excessive inflammation and collateral tissue damage. In particular, zinc impacts macrophage function, a critical front-line cell in host defense, in addition to other immune cells. Deficits in zinc adversely impact macrophage function resulting in dysregulation of phagocytosis, intracellular killing, and cytokine production. An additional work in this field has revealed a vital role for several zinc transporter proteins that are required for proper bioredistribution of zinc within mononuclear cells to achieve an optimal immune response against invading microorganisms. In this review, we will discuss the most recent developments regarding zinc's role in innate immunity and protection against pathogen invasion.

## 1. Zinc and Human Health

Zinc (Zn) is an essential micronutrient in human health. According to the World Health Organization (WHO), an estimated 30% of the world's population is Zn deficient and inadequate intake contributes to 800,000 deaths worldwide [1]. Although severe Zn deficiency is rare, mild-to-moderate deficiency is prevalent throughout the world [2, 3]. It is the fifth leading risk factor for bacterial acquired diarrhea and pneumonia in developing countries [4]. These findings underscore the importance of Zn deficiency as a well-established nutritional problem in underdeveloped countries; however, it is important to recognize that Zn deficiency is also common in developed countries. According to the National Health and Nutrition Examination Survey (NHANES) that is conducted within the United States (US) population, the percentage of dietary intakes below the estimated average daily requirement is quite common in the elderly and up to 24% in 14- to 18-year-old females and 12% in adults over 19 years of age. Collectively, this indicates that inadequate Zn intake is quite common and may contribute substantially to comorbidity throughout the world across diverse populations.

Zinc is essential for the proper function of both eukaryotic and prokaryotic cells. It is required for normal growth, repair, and maintaining the structure and function of proteins and nucleic acids including enzymes and transcription factors. Despite the vital necessity of Zn, there exist no depots of Zn in the body that can be used to maintain metal levels for protracted periods of time [5]. This is further complicated in developing countries where other factors including limited protein-based food choices rich in bioavailable Zn significantly perpetuate Zn deficiency [6, 7]. A major contributing factor to this dilemma results from higher consumption of phytates, an inhibitor of Zn absorption in the gastrointestinal tract [8]. In addition, intestinal malabsorption, renal disease, alcoholism, cancer, aging, pharmacologic interactions, and alteration in Zn transporters can contribute to Zn deficiency [9, 10]. Further, the elderly, infants, and pregnant and lactating women are more susceptible to Zn deficiency and corresponding infections that can be fatal if not corrected with dietary supplementation [11, 12].

This evidence substantiates that dietary Zn uptake is important in maintaining homeostasis, proper innate immune balance, and host defense [13]. Zn also possesses known anti-inflammatory and antioxidant effects, and nutritional supplementation has been reported to be beneficial in suppressing the duration and severity of infectious-based gastrointestinal and respiratory disease particularly in children less than 5 years of age [14]. Given the many roles of Zn in maintaining immune function, it is not surprising that Zn deficiency increases the risk of morbidity and mortality from infection.

## 2. Zinc and Infection in Mammals

Zn is essential for the proper function of the innate and adaptive immune systems; therefore, depletion of whole body Zn content adversely impacts the ability of the host to mount a balanced immune response against invading pathogens [15]. Multiple epidemiologic studies conducted over the past two decades demonstrate that Zn deficiency increases susceptibility to infections that the host would otherwise, under normal circumstances, be less vulnerable to. Most notably inadequate Zn intake is associated with new-onset upper respiratory tract and gastrointestinal tract infections in children less than 5 years old in developing countries [16, 17]. More recently, it has been shown that gastrointestinal giardiasis impairs intestinal mucosa function thereby lowering Zn absorption in young children resulting in Zn deficiency [18]. Given the importance of Zn relative to host defense, multiple studies have been conducted in humans and animals to determine whether Zn supplementation can restore immune function and prevent or reduce the severity of infection. Zn intake was originally shown to reduce the incidence of acute lower respiratory infection in preschool children [19]. Sánchez and colleagues more recently reported that the incidence of acute diarrhea and respiratory infection was reduced following the administration of a Zn amino acid chelate in children [20]. Sazawal and colleagues reported that Zn supplementation reduced duration and severity of diarrhea in infants and young children [21], and Prasad and colleagues reported that the incidence of bacterial infection in sickle cell disease was significantly reduced after Zn supplementation [22]. Bolick and colleagues reported that Zn-deficient diet mice had significantly greater weight loss and diarrhea compared to control mice when infected with enteroaggregative *Escherichia coli* (EAEC), a pathogen that is responsible for infectious diarrhea in children. In addition, Zn-deficient mice had reduced infiltration of leukocytes into the ileum in response to infection indicative of an impaired immune response [23]. Similarly, mice infected with HlyA-producing *E. coli* were able to better maintain epithelial function and reduce barrier disruption and fluid leakage with diminished bacterial translocation as a result of Zn supplementation [24]. More recently, administration of Zn oxide nanoparticles was shown to reduce fluid accumulation in the mouse ileum following the administration of cholera toxin protein [25]. Zinc supplementation has also been reported to be beneficial in animal models of severe infection. Sepsis mortality was significantly increased in Zn-deficient animals when compared to animals maintained on a Zn sufficient, control diet subject to cecal ligation puncture. Prior to death, Zn-deficient animals exhibited higher plasma cytokines, oxidative tissue damage, cell death, and more severe vital organ injury. However, when Zn supplementation was provided to Zn insufficient mice prior to the initiation of sepsis, it resulted in the normalization of the inflammatory response, diminished tissue damage, and significantly reduced mortality [26]. Zn supplementation has most often been reported to be beneficial for restoring immune function and downregulating conditions associated with chronic inflammatory responses [27]. Zn supplementation has also been shown to improve macrophage phagocytosis and oxidative burst function [28] and reduce C-reactive protein, IL-6, MMP2, and MMP9 production in a model of chronic liver inflammation [29]. Zn is also crucial for maintenance and function of the immune system during the process of aging [30]. Unfortunately, human studies where Zn supplementation has been provided to critically ill patients have not yet proven to be beneficial suggesting that critical illness is more complicated to treat [31]. Based on this, well-controlled, large, randomized control trials are required to determine whether optimal Zn dosing strategies exist. Notably, sepsis creates a significant challenge because it is difficult to anticipate the onset of sepsis thereby prohibiting preventive treatment strategies. Collectively, these studies strongly support the importance of Zn as an essential nutrient in host defense against pathogen invasion in the lung, gut, and systemic compartments and also highlight the need for more study aimed at determining how best to administer Zn to populations that are more susceptible to infection.

## 3. Zinc and Myeloid Cell Function against Pathogens

Invading pathogens are first encountered by cells that comprise the innate immune system. Myeloid lineage cells including polymorphonuclear neutrophils, monocytes, and macrophages are critical first responders engineered to recognize and eliminate pathogens. Micronutrient metabolism plays a critical role in innate immune defense against microbial infection. Macrophages exploit transition metals in part by manipulating their uptake and trafficking following pathogen recognition. Cation redistribution into the cytosol, in general, is designed to benefit the host in a number of important ways. It inhibits pathogen growth through deprivation of indispensable micronutrients, generates host protective Fenton reaction-dependent reactive oxygen species, and affords nonspecific inhibition of bacterial protein binding [32–34]. Importantly, internalized micronutrients also help orchestrate vital signaling pathways. Macrophages differ significantly from monocytes in their phenotype and function and the metabolic pathways responsible for zinc trafficking during macrophage host defense remain largely unexplored [33, 35]. In response to microbes, macrophages produce both proinflammatory cytokines and anti-inflammatory cytokines, like IL-10, in order to coordinate a balanced response aimed at efficiently eliminating infection while minimizing damage to surrounding tissue [36]. Importantly, IL-10 stimulation of murine and human macrophages significantly reduces production of proinflammatory cytokines [37–40]. Zn has been shown to regulate endotoxin-mediated immune activation of human macrophages through a reduction in IL-10 production presumably leading to imbalance in immune regulation, although the latter was evaluated in this study [41]. In yet another mechanism of Zn sequestration, Histoplasma capsulatum induced mobilization of Zn within macrophages away from the phagosome and into the apparatus triggering superoxide formation and enhancing antimicrobial defense [42]. In the case of Salmonella infection, a bacteria-driven elevation of intracellular Zn levels occurs weakening antimicrobial defense and the ability of macrophages to eradicate the pathogen thereby demonstrating that certain bacteria can establish host subversion strategies that use Zn to their advantage [43, 44]. Based on these findings, it is clear that there exists a tug-of-war between macrophages and other myeloid-lineage cells and the pathogens that they encounter. In the case of bacteria that reside and thrive outside of macrophages, cellular activation most often leads to Zn-mediated changes that favor the host. In contrast, intracellular pathogens have the potential to subvert Zn metabolism and cellular activation in a manner that may favor pathogen survival and propagation within the macrophage.

Zinc is also important for monocyte and macrophage development and other vital functions including phagocytosis and cytokine production [45, 46]. Zinc is also necessary for adherence, migration, and differentiation of monocytes into tissue macrophages [47] and is crucial for the normal development and function of neutrophils. Zinc deficiency causes dysfunctional neutrophil phagocytosis, intracellular killing, and cytokine production [10]. In addition, neutrophil chemotaxis and the quantity of enzyme-containing cytosolic granulocytes decrease as a result of Zn deficiency [48]. A major role of neutrophils is to clear invading pathogens through formation of an extracellular network trap that is also disrupted under Zn-deficient conditions [49]. Therefore, recent studies have identified that intracellular Zn content within myeloid-lineage cells is absolutely critical in maintaining a defensive phenotype in a number of ways that influence organelle and protein function in order to enhance bacterial elimination.

Multiple cell signaling pathways are influenced by intracellular free Zn levels [50]. One of the most studied is the NF-*ĸβ* signaling pathway. In particular, insufficient levels of intracellular Zn result in aberrant cell signaling causing an exaggerated inflammatory response as exhibited by the overproduction of cytokines and chemokines including TNF*α*, IL-6, IL-8, and IL-10. Zinc deficiency can also induce changes in the expression of other cytokines, DNA repair enzymes, zinc transporters, and signaling molecules that regulate immune function. As one example, macrophages have an armamentarium of recognition receptors, including Toll-like receptors (TLR), designed to recognize bacterial, viral, and fungal byproducts. Endotoxin is one of the most robustly studied ligands for TLR4 signaling. Transmission of cell signaling in monocytes is Zn dependent following LPS recognition and impact activation of the p38 MAPK, ERK1/2, and NF-*κβ* pathways [51]. Depletion of Zn was also shown to inhibit endotoxin-induced activation of several MAPK kinase family members and I*κ*B*α* in murine primary macrophages and corresponding cell lines [52]. In a polymicrobial sepsis mouse model, Zn deficiency was shown to enhance bacterial burden, NF-*κβ* activity, and the corresponding IL1*β*, TNF*α*, and ICAM-1 gene expression and protein production in the lung when compared to Zn sufficient control animals [53]. Another study by this group revealed that Zn deficiency enhanced signaling through the JAK-STAT3 pathway and NF-*κβ* pathway leading to increased inflammation and over activation of the inflammatory response in a polymicrobial sepsis mouse model [54]. Similarly, Zn has been shown to be critical in regulation of kinase activation and transcription factors that subsequently alter the expression of proteins [55]. Zinc has also been reported to alter the function of different immunologically relevant mitogens and bacterial stimulants. As one example, Zn was shown to enhance the immunogenicity of lipopolysaccharide with respect to cell recognition and cytokine induction in leukocytes by altering its structure into a more biologically active isoform [56].

Collectively, Zn-mediated regulation of innate immune host defense through myeloid lineage cells demonstrates that Zn is absolutely required to maintain proper function. In particular, deficits in Zn that typically occur across many populations as a result of insufficient dietary intake and or absorption result in dysregulated myeloid cell function in response to invading pathogens. The extent of immune dysfunction is context dependent in terms of host phenotype (age, gender, and health status), location of the infection (lung, gut, bloodstream), and cell type that pathogens encounter in these compartments (neutrophils, monocytes, macrophages, and other myeloid cell types not yet studied), as well as the pathogen (bacterial, viral, and fungal). A key aspect of Zn, beyond having sufficient amounts in our body, involves its rapid and controlled mobilization from the bloodstream into tissues and ultimately into cellular compartments and organelles where it has a direct impact on enzymatic function. In this regard, mammals have developed a sophisticated system to shuttle Zn in and out of cells and cellular organelles, and into specific locations within the cell allowing Zn to specifically interact with nucleic acids and proteins in a manner that positively impacts cellular function and bolsters host defense.

## 4. Zinc Transporters

The concentration of cellular Zn is rather high such that Zn can hardly be considered a trace element. Zinc is used as a cofactor in proteins much more frequently than most vitamins. Control over the fluctuating Zn pool occurs at remarkably low concentrations, often within the picomolar range, through the participation of many proteins [57]. Zinc homeostasis in mammals is primarily maintained through Zn transporters that are designed to regulate cellular uptake, efflux, and intracellular trafficking of Zn. The majority of intracellular Zn exists in a tightly bound form most commonly associated with metalloproteins and Zn finger proteins. Approximately, ten percent of cellular Zn exists in a more loosely bound form commonly referred to as labile Zn. Whereas the tightly bound form of Zn is not subjected to rapid mobilization, the labile pool can be mobilized within and outside of cells very rapidly in response to cellular stress and infection. The labile pool therefore plays an important role in intracellular Zn fluxes and rapid alteration of cell function, but it is also more readily depleted in the setting of Zn deficiency [58]. There exist two major Zn transporter/carrier families known as (solute carrier family = SLC) SLC30 and SLC39 that have been extensively studied for their roles in maintaining cytosolic as well as cellular organelle Zn levels. There are 10 members of the human SLC30^1–10^ or ZnT family that are primarily involved with transporting zinc from the cytosol to the extracellular space or into intracellular organelles and therefore function largely to efflux Zn out of the cytosol especially in conditions of excess [59]. The SLC39 family or ZIP (Zrt-, Irt-like proteins) is comprised of 14 members that are primarily involved in transporting Zn from the extracellular space or intracellular organelles into the cytosol and therefore function to increase cytosolic zinc concentrations [60]. ZIPs and ZnTs are involved in many cellular responses. Through the mobilization of Zn, they regulate enzymes, receptors, and transcription factors as well as cytokine and growth factor-mediated signaling pathways [61]. In collaboration with metallothioneins (MTs), these proteins collectively maintain cellular Zn content thereby countering rapid or prolonged changes in available Zn in a manner designed to maintain normal cellular function yet maintain cells in a vigilant state in advance of potentially harmful external challenges [62, 63].

Cytosolic labile Zn content can change rapidly in response to pathogens and plays an important role in Zn signaling that triggers immune activation [64]. Upon extracellular stimulation, Zn is rapidly released as a “zinc wave” into the cytosol from intracellular stores. This phenomenon activates two mitogen-activated protein kinase (MAPK) pathways, the extracellular signal-related kinase (ERK) and the c-Jun N-terminal kinase (JNK) signaling pathways [60] and the transcription factor NF-*κ*B, which targets genes involved in cellular replication and apoptosis [65]. The expression and function of certain Zn transporter genes are responsive to physiologic stimuli and external challenges, whereas others appear to be constitutively expressed and vital for the day-to-day maintenance of body Zn composition. Moving forward, we will focus on Zn transporters that have been shown to be responsive to pathogen invasion and host defense. A variety of stimuli, including inflammatory cytokines and pathogens or bacterial byproducts, have been shown to induce the expression of MTs, ZnTs, and ZIP transporters [66]. Importantly, altered expression of Zn transporters during inflammatory conditions is part of the acute phase response that is designed to rapidly mobilize labile Zn in a manner that affords cell protection, maintains or increases vital functions, and bolsters host defense [63]. As a leading example, ZIP14 (slc39A14) was the first Zn transporter shown to be highly upregulated in the liver, white adipose tissue, and muscle in a mouse model after exposure to endotoxin LPS [67]. Studies conducted in a mouse model of endotoxemia first revealed that ZIP14 expression is upregulated through IL-6 and that this Zn transporter plays a major role in the mechanism responsible for hypozincemia, not to be mistaken with Zn deficiency as a result of insufficient dietary intake that accompanies the acute-phase response to inflammation and infection [68]. This group went on to reveal that ZIP14 plays an important role in LPS-mediated IL-6 release and that IL-6 synthesis after LPS stimulation is regulated by STAT3 and I*κ*B phosphorylation. However, in LPS-injected Zip14 KO mice, mRNA expression of suppressor of cytokine signaling-3 (SOCS-3), a downstream target gene of the STAT3-I*κ*B pathway, was markedly increased in LPS-injected null mice [67]. A related study showed that following intraperitoneal administration of lipopolysaccharide or the proinflammatory cytokines tumor necrosis factor (TNF) or interleukin-6 (IL6), mice exhibited quantitatively very different, highly tissue-specific, and markedly time-dependent up- and downregulation of ZIP8 and ZIP14 mRNA levels in vital organs. This revealed for the first time that most if not all tissues use ZIP8 and ZIP14 to some extent for Zn uptake. Some tissues are under basal conditions and others more so when inflammatory stressors are present that collectively lead to substantial alterations in plasma Zn levels due to Zn redistribution not just in the liver but across many vital organs [69]. More recently, using *Zip14* knockout (KO) mice, it was shown that ablation of *Zip14* delayed leukocytosis, prevented zinc accumulation in the liver, altered the kinetics of hypozincemia, and drastically increased serum IL-6, TNF*α*, and IL-10 concentrations following nonlethal sepsis further establishing that ZIP14 is vital to host defense in the setting of polymicrobial sepsis [70]. Consistent with these observations, the closest homologue to ZIP14, ZIP8, was shown to have induced expression in lung cells in response to LPS or TNF-*α* resulting in the expression of a heavily glycosylated, membrane-bound protein that lead to Zn import and cell survival [71]. A further work by this group revealed that ZIP8 plays a vital role in regulating the host response to sepsis, in part through monocyte and macrophage regulation, by downmodulating the NF-*ĸβ* pathway. In particular, ZIP8 gene expression was induced by the NF-*ĸβ* pathway following bacterial activation and the new pool of Zn transported into myeloid cells ultimately inhibited IKK*β* activation [41]. Relative to myeloid-lineage cells, ZIP8 expression was observed to be elevated in macrophages following LPS exposure resulting in cellular accumulation of Zn and enhanced IL-10 release. Further, ZIP8 was vital to immune function because knockdown inhibited LPS-driven Zn accumulation and reduced Zn-dependent reduction of IL-10 release [41]. Similarly, *Mtb* infection of macrophages was shown to induce the expression of ZIP8 with a very little effect on other ZIPs although it remains to be determined whether ZIP8 enhances Zn uptake in favor of the host or pathogen [72]. Taken together, Zn transporter-mediated Zn redistribution via ZIP8 and ZIP14 upon infection likely serves multiple purposes [73]. In addition to these two closely related homologues, other Zn transporters have more recently been shown to play a role in host defense as well. In particular, a recent study using macrophage-specific *Slc39a10*-knockout mice, we revealed that Slc39a10 plays an essential role in macrophage survival by mediating Zn homeostasis in response to LPS stimulation. Compared with *wild-type* mice, *Slc39a10*-knockout mice had significantly lower mortality following LPS stimulation as well as reduced liver damage and lower levels of circulating inflammatory cytokines [74]. Based on these findings, a picture is beginning to emerge that multiple Zn transporters play important roles in the strengthening innate immune function, in part by regulating macrophage behavior, in response to systemic bacterial invasion.

In the last decade, mutations in ZIP and ZnT transporter genes have been shown to be implicated in a number of inherited human diseases many of which lead to inflammation-based pathology and therefore may have relevance to infectious-based disease [75]. Moreover, dysregulation in the expression and activity of both transporter families has been suggested to be involved in the pathogenesis and progression of multiple diseases [76, 77]. As leading examples, mutations in ZIP4 (SLC39A4) and ZIP13 (SLC39A13) are responsible for the rare lethal autosomal-recessive inherited zinc deficiency disease, acrodermatitis enteropathica (AE) [78], and Ehlers-Danlos Syndrome (EDS) [79], respectively. Additionally, targeted ZnT3 disruption causes memory deficits with Alzheimer's disease-like abnormalities in mice [80]. Another study reported that a polymorphic genetic variant of ZnT10 was related to hypermagnesemia as well as a variety of neurological and hepatic disturbances [81]. Abnormal hematopoiesis and organ morphogenesis have been reported in ZIP8-hypomorphic mice [82]. Collectively, these studies strongly support the importance of the normal expression and function of Zn transporters in order to maintain Zn homeostasis in mammals but how this impacts the host response to pathogen invasion remains to be determined. In all likelihood, we predict that commonly occurring polymorphic variants of Zn transporters, including but not limited to ZIP8 and ZIP14, have the potential to increase susceptibility to infection by adversely altering Zn metabolism during the acute-phase response and thereby altering cellular defense mechanisms in myeloid and other parenchymal cell types.

## 5. Conclusion

Zinc is an essential metal that plays an important role in maintaining mammalian health and fending off invasive pathogens. It is fundamentally important in maintaining biochemical balance and influences multiple components of the innate immune system through modulation of protein function. In developing countries, limited uptake of Zn-containing foods, often a consequence of low economic status, has provided the best evidence that a lack of dietary Zn intake is a major contributing factor in susceptibility to bacterial infections. In recent years, using cell and animal models, it has become clear that the increased morbidity and mortality associated with Zn deficiency and bacterial infection are a consequence of impaired immune regulation. Zinc and a select number of Zn transporters have begun to emerge in recent years as a novel host defense strategy aimed at quickly mobilizing labile Zn within the body to, in most instances, bolster host defense and sequester Zn away from prokaryotic cells that also require Zn to maintain vital functions. Although exactly how Zn metabolism regulates, host defense will require much more study because there remain many unanswered questions. Additional studies will be required to better understand the multiple roles of Zn relative to innate immune signaling across different cell types, including but not limited to myeloid cells, through initiation and maintenance of an effective host response. What does seem clear is that a Zn deficit in most cases leads to exaggeration of the host defense response against pathogens. While at face value it would appear that amplification of host defense mechanisms would be advantageous, this is often not the case. In fact, exaggeration of the host response to infection has most commonly been shown to increase the risk of collateral tissue damage leading to poorer prognosis in terms of morbidity and mortality. To better understand the role of Zn transporters in pathogen-related pathological conditions, additional investigations of the molecular mechanisms of Zn-mediated regulation of immune function are warranted. In particular, some Zn transporters are known to transport other metal ions besides Zn; however, a number of these studies are currently limited and have primarily focused on the relative uptake affinity and biodistribution for other metal ions including iron, cadmium, and manganese. Whether these metals also play an important role in maintaining proper host defense against pathogens remains to be determined. In addition, this could lead to the design and synthesis of new compounds directed toward therapeutic targets that either modulate protein function or alter metal uptake and efflux via Zn transporters. Finally, a limited number of important studies have provided proof of principal that Zn supplementation can decrease the incidence and severity of bacterial infections in populations where the frequency of Zn-deficient intake is high. Most importantly, Zn has been shown to be the most beneficial when used as a preventive strategy prior to the development of established infection. In all likelihood, there does not exist a one size fits all therapeutic approach. Given the substantial biochemical footprint of Zn across the human proteome, it is also plausible that Zn supplementation could be disadvantageous under certain conditions against different pathogens. Perhaps most importantly, strategies that more accurately detect subacute Zn deficiency will expedite our ability to predict and prevent serious, life-threatening infection. Past studies have largely focused on the utility of blood and tissue Zn levels or protein biomarkers, with limited success. As an alternative approach, the genetic heterogeneity of Zn transporters relative to increased disease risk has begun to emerge and may provide a predictive tool to identify at-risk populations against different pathogens based on screening for polymorphic genetic variants that alter normal Zn metabolism and immune function. Scientific advances in the field of Zn biology coupled to advances in high throughput genetic and proteomic screening approaches will undoubtedly shed new light on this emerging field and hopefully provide translational advances that improve our capacity to predict and prevent the extent of infectious disease in many different settings across vast populations.
